# Electrospun Nanofiber Membranes with Various Structures for Wound Dressing

**DOI:** 10.3390/ma16176021

**Published:** 2023-09-01

**Authors:** Jiahao Yang, Lan Xu

**Affiliations:** 1 National Engineering Laboratory for Modern Silk, College of Textile and Engineering, Soochow University, 199 Ren-Ai Road, Suzhou 215123, China; 20224215045@stu.suda.edu.cn; 2 Jiangsu Engineering Research Center of Textile Dyeing and Printing for Energy Conservation, Discharge Re-Duction and Cleaner Production (ERC), Soochow University, Suzhou 215123, China

**Keywords:** electrospinning, nanofiber membrane, material, structure, wound healing

## Abstract

Electrospun nanofiber membranes (NFMs) have high porosity and a large specific surface area, which provide a suitable environment for the complex and dynamic wound healing process and a large number of sites for carrying wound healing factors. Further, the design of the nanofiber structure can imitate the structure of the human dermis, similar to the natural extracellular matrix, which better promotes the hemostasis, anti-inflammatory and healing of wounds. Therefore, it has been widely studied in the field of wound dressing. This review article overviews the development of electrospinning technology and the application of electrospun nanofibers in wound dressings. It begins with an introduction to the history, working principles, and transformation of electrospinning, with a focus on the selection of electrospun nanofiber materials, incorporation of functional therapeutic factors, and structural design of nanofibers and nanofiber membranes. Moreover, the wide application of electrospun NFMs containing therapeutic factors in wound healing is classified based on their special functions, such as hemostasis, antibacterial and cell proliferation promotion. This article also highlights the structural design of electrospun nanofibers in wound dressing, including porous structures, bead structures, core-shell structures, ordered structures, and multilayer nanofiber membrane structures. Finally, their advantages and limitations are discussed, and the challenges faced in their application for wound dressings are analyzed to promote further research in this field.

## 1. Introduction

Skin, as the largest organ of the human body, can regulate body temperature and resist the invasion of various pathogens and microorganisms [1], a natural barrier for the internal environment of the human body to directly contact the outside world. Therefore, skin integrity is very important for human health [2]. Wounds can be caused by the injury and destruction of skin tissue, and many accidents in daily life can easily lead to wounds. After trauma, the skin recovery process is dynamic and complex, including four stages: hemostasis, inflammation, cell proliferation and remodeling [3]. Moreover, special diseases such as diabetes also affect the wound microenvironment and extend its healing time. According to the difference in wound recovery time, wounds can be divided into acute and chronic. For chronic wounds, the recovery time is long, and the risk of exogenous negative interference is high. To protect wounds from external pollution, avoid wound deterioration and promote wound healing, the use of wound dressings is very critical for wound treatment. Due to the complex and dynamic process of wound healing, wound dressings need good biocompatibility, stability, certain mechanical properties, permeability, and the ability to absorb the excess tissue osmotic fluid produced by the wound. Further, the ideal wound dressing should also have antibacterial and anti-inflammatory functions to promote cell growth and accelerate wound healing [4].

Wound dressings have a long history, dating back to 1550 BC, when wound dressings were a mixture of oil, honey, and cotton wool. With the continuous expansion of research, multifunctional dressings that can provide an ideal wound recovery environment are applied to wound healing. At present, the main forms of wound dressings are gauze, bandages, sponges [5], films [6], scaffolds [7], hydrogels [8] and nanofibers, among which gauze and bandages are traditional dressings with low-cost performance, but their effects on wound healing are limited to protecting the wound from external stimulation, and there is a risk of secondary injury caused by adhesion to the wound. Sponge, hydrogel, and nanofiber dressings are new dressings developed by researchers according to the characteristics of wound healing to accelerate wound healing. Compared with these wound dressings, nanofibers have significant advantages, such as high permeability and specific surface area.

Meanwhile, nanofibers can also form a structure similar to the natural extracellular matrix (composed of interwoven protein fibers), providing a favorable environment for the adhesion and proliferation of cells and promoting the transport of nutrients. Moreover, some studies have shown that cells adhere to fibers smaller in diameter than themselves [9]. The rapid development of nanotechnology positively impacts the preparation of nanofibers. The preparation methods of nanofibers mainly include melt blowing, rotary jet spinning, manual spinning, pressurized rotary spinning and electrospinning [10,11,12,13], which have been developed for manufacturing drug-loaded nanofiber scaffolds.

Electrospinning is a low-cost, simple, and flexible process for producing nanofibers. Nanofibers prepared by electrospinning technology have strong programmability, and the nanofibers with controllable structure and uniform continuity can be fabricated by adjusting the preparation process parameters [14], which makes them widely used in catalysis [15], filtration [16], electrochemistry [17] and food engineering [18]. Furthermore, the structure and composition of electrospun NFM can be similar to those of the natural extracellular matrix, and their high porosity can promote the attachment, migration and proliferation of cells [19], which makes them have great potential in the fields of biosensors [20], drug transportation [21] and wound dressing [22]. Especially as wound dressings, electrospun NFMs with high surface area and high porosity can provide a good environment for the exchange of water and gas between the wound surface and the outside world, which is conducive to the absorption of tissue osmotic fluid at the wound and the carrying of therapeutic factors [23]. Moreover, the inherent high flexibility and toughness of NFMs provide convenience for using different parts of wounds. Meanwhile, the design and combination of materials and structures for electrospun nanofibers can also make them more likely to simulate the structure and function of natural skin and promote wound healing [24].

In recent years, there have been many reviews based on electrospun nanofibers for wound dressings. However, there are few reviews on the effects of the structural design of electrospun nanofibers used for wound dressings on wound recovery. Therefore, this review provides an overview of the development and working mechanism of electrospinning technology and discusses various process parameters affecting nanofibers, with a focus on a selection of electrospun nanofiber materials, incorporation of functional therapeutic factors, and structural design of nanofibers and nanofiber membranes. Further, the recent progress of electrospun NFMs containing therapeutic factors in wound dressings is classified according to their specific functions, such as hemostasis, antibacterial, and cell proliferation promotion. This article also highlights the structural design of electrospun nanofibers in wound dressing, including porous structures, bead structures, core-shell structures, ordered structures, and multilayer NFM structures. Finally, their advantages and limitations are discussed, and the challenges faced by electrospun NFMs for wound dressings are analyzed to promote further research in this field.

## 2. Electrospinning Technology

As early as 1600, William Gilbert discovered the electrostatic motion of liquids. In 1887, Charles Vernon and his team reported the extraction of fiber from liquid under an applied electric field [25]. In 1964, Taylor proposed the effect of current on the conical formation of solution droplets using mathematical modelling. Morton and Cooley put forward the first patent on electrospinning technology in 1902 [26]. After that, Formhals applied for several patents on this technology, and electrospinning technology has been greatly developed and gradually matured [27]. The important developmental stages of electrospinning are described in detail in Ref. [28].

**Figure 1:**
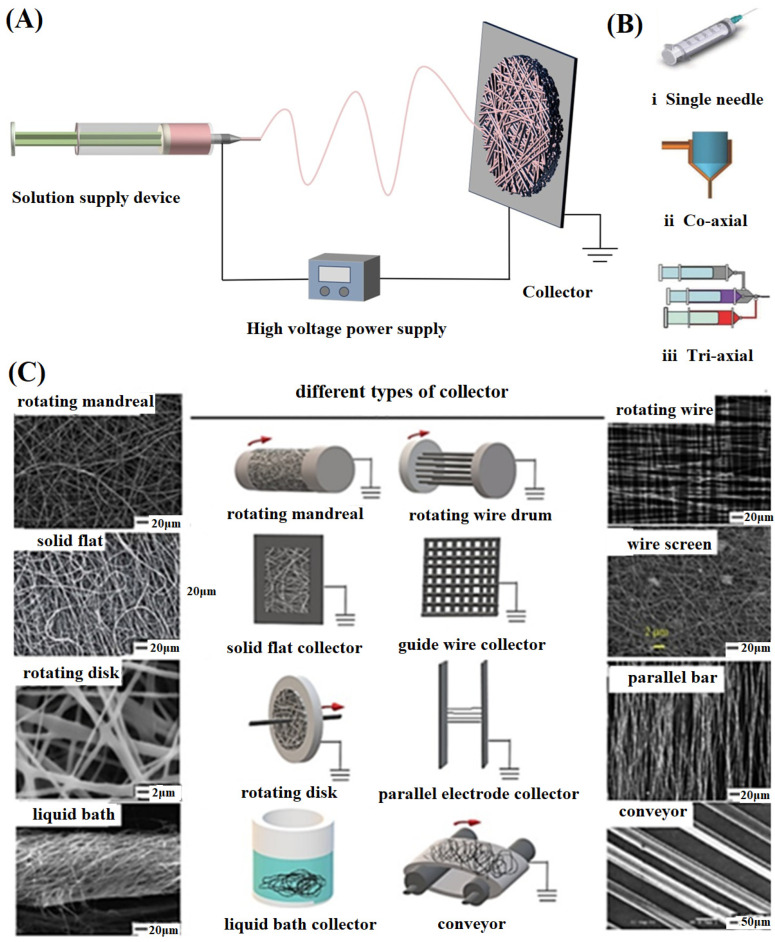
(**A**) Schematic diagram of the electrospinning process; (**B**) Various types of needles reproduced with permission from [31]; (**C**) Different types of collectors reproduced with permission from [26].

Electrospinning involves the stretching of liquid droplets under the action of an electric field force and elongation into fibers. As shown in Figure 1A, the traditional electrospinning device consists of a liquid supply device with a spinneret, a high-voltage power supply and a collector. The high-voltage power supply connects the liquid supply device and the collector, forming a strong electric field. When the polymer solution or melt is pushed out of the spinneret under a certain pressure, liquid droplets will form at the nozzle. Meanwhile, the liquid droplets subject to the combined action of surface tension and electrostatic force will be deformed into conical liquid droplets, also known as a “Taylor cone” [29]. When the voltage exceeds a certain threshold value, the force on the surface of the droplet is unbalanced, and then a charged jet is ejected at the tip of a conical droplet. During the jet movement, the solvent evaporates rapidly in the air, and then the solute solidifies into a fiber. The instability of solvent evaporation and the change in electrostatic force can lead to the jet’s bending and irregular movement of the jet, and finally, the jet forms fibers with diameters ranging from microns to nanometers randomly deposited on the collector [30].

The morphology, diameter, porosity, and mechanical properties of electrospun nanofibers can be regulated by selecting suitable spinning parameters. The parameters mainly include process parameters, solution parameters and environmental parameters. Process parameters generally refer to voltage [32], flow rate, and distance between the spinneret and collector. Spinning solution parameters mainly contain polymer molecular weight [33], viscosity, surface tension, concentration, conductivity, etc. The environmental parameters are primarily temperature and humidity. These parameters interact in a certain range and jointly affect the morphology of electrospun nanofiber, as summarized in Table 1.

Currently, many types of electrospinning equipment have been designed and developed. Based on traditional electrospinning, the emergence of coaxial electrospinning supports the preparation of nanofibers with a core-shell or multi-layer structure to meet special needs, as shown in Figure 1B. Moreover, coaxial electrospinning can reduce its requirements for spinning solutions, and the obtained multilayered nanofibers provide better protection for carrying biologically active factors, avoiding the sudden release of drugs in the blend fibers. In addition, the shape and motion of the collector can be adjusted to control the morphology and arrangement of nanofibers, as shown in Figure 1C. The design of electrospinning needles and the modification of collectors provide more possibilities for the structure and morphology of nanofibers, making them similar to human tissue structures [44].

**Figure 2:**
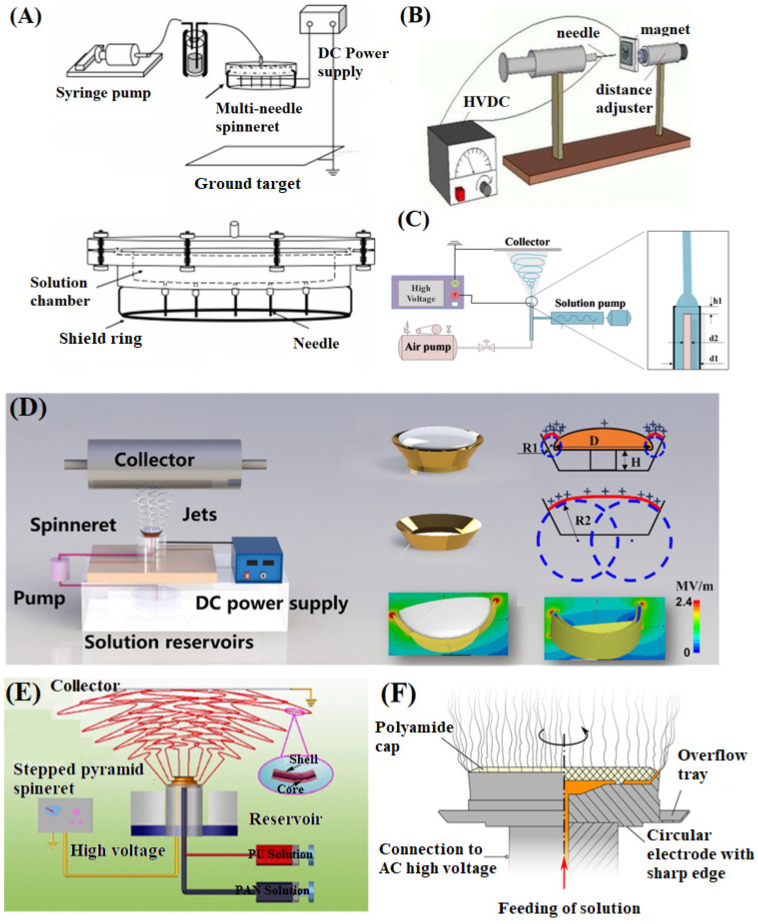
(**A**) Schematic of an electrospinning apparatus with an auxiliary electric field, reproduced with permission from [46]; (**B**) Schematic of an electrospinning apparatus with magnetic field, reproduced with permission from [51]; (**C**) Schematic of an electrospinning apparatus with airflow, reproduced with permission from [52]; (**D**) Schematic illustrations of mushroom-electrospinning setup, red color symbols represented the surface curvature and surface charge distribution of the covered and uncovered mushroom-spinneret and the blue circles were to help understand them, reproduced with permission from [54]; (**E**) Scheme of the free surface coaxial electrospinning apparatus using a stepped pyramid spinneret [55]; (**F**) Schematic of an electrospinning apparatus with centrifugal force, reproduced with permission from [57].

Further, the electrospinning efficiency is effectively improved through the design and improvement of the spinneret, and many effective strategies have been proposed, such as increasing the number of needles/nozzles and using needleless electrospinning [45]. Increasing the number of needles is a simple and effective method to increase nanofiber production, where the specification, position, number, and arrangement of needles all affect the distribution of electric fields, thereby affecting the distribution of deposited fibers. Therefore, multi-needle electrospinning requires large operating space and appropriate needle spacing to avoid charge repulsion between jets. The common needle arrangements include straight lines, squares, circles, triangles, and hexagons [46,47]. For a given needle holder configuration and configuration angle, the electric field remains constant regardless of the number of needles [48]. In addition, multi-needle electrospinning can deposit nanofibers of different materials on the collector to enhance the performance of the prepared composite NFM [49]. The electrostatic repulsion between needles can be effectively alleviated by adjusting the distance between them, which is determined by the electric field intensity and the properties of the spinning solution [50]. Moreover, the use of auxiliary electric fields (Figure 2A), magnetic fields (Figure 2B), and airflow (Figure 2C) can control the direction of jet motion, thereby constraining the stability of jets as well as the size and arrangement of fibers [46,51,52].

Compared with multi-needle electrospinning, needle-free electrospinning requires higher voltage to overcome the surface tension of the liquid and stimulate the formation of multiple jets, which can effectively solve the problem of needle plugging [53]. Xiong et al. [54] designed a mushroom-shaped electrospinning device that avoided the appearance of competitive electric fields, promoted the formation of stable circular pre-Taylor cones with high curvature, and significantly improved production efficiency (Figure 2D). Molnar et al. [55] improved the free surface electrospinning device, which had a narrow and long liquid supply slot and sharp edges (corona) from the metal electrode ring. The Taylor cone was formed on a sharp edge with high charge density, and the spinneret could rotate at an adjustable speed, thus effectively reducing solvent volatilization and achieving the production of high-throughput nanofibers. Jiang et al. [56] proposed improved needle-free electrospinning that could produce core-shell fibers on a large scale by using a stepped pyramid-shaped copper spinneret (Figure 2E). The coaxial jet was generated from the pyramid-shaped edge, avoiding the problem of easy blockage in the coaxial needle and significantly improving the production speed of core-shell fibers. Farkas et al. [57] combined centrifugal force and needle-free electrospinning for batch preparation of nanofibers, achieving the regulation of nanofiber diameter, which greatly enriched the development of electrospinning technology (Figure 2F). 

## 3. Materials

### 3.1. Matrix Materials

Polymers are the main matrix materials for electrospun nanofibers, which can be divided into natural polymers and synthetic polymers. Natural polymers have the advantages of good biocompatibility, friendly structure, and nontoxicity, which make them popular materials in the biomedical field. Meanwhile, it has been proven that materials with good biocompatibility and degradability can be recognized by cell surface receptors in wound recovery, thus causing cell adhesion and proliferation [58]. Common natural polymers include polysaccharides (such as chitosan, cellulose, hyaluronic acid, and alginate) and proteins (such as collagen, silk fibroin, gelatin and fibrin).

Chitosan has become a vital member of the wound dressing material family due to its excellent biological and antibacterial properties [59]. In the inflammatory stage of wound recovery, chitosan can promote the migration of cells to the wound area, which is conducive to the elimination of microorganisms by macrophages, decomposition of dead cells, and stimulation of cells related to tissue and angiogenesis. However, chitosan has poor solubility and is difficult to directly electrospun [60]. A common strategy is to prepare chitosan-based electrospun nanofibers by controlling chitosan’s molecular weight and deacetylation degree or blending with other materials [61,62]. Cellulose has excellent thermal stability, chemical resistance, and good biological properties, which can reduce pain and promote the formation of granulation and epithelization at the wound. Due to the limited solubility of cellulose, cellulose acetate based on modified cellulose is mostly used to prepare nanofibers [63,64]. In addition, bacterial cellulose has good biological function and excellent mechanical, which has a positive impact on vascular regeneration, damaged tissue remodelling and wound healing [65]. Collagen is a three-dimensional network structure composed of nanoscale fibrils and extracellular matrix proteins [66]. Therefore, electrospun nanofibers prepared with collagen are very similar to the natural extracellular matrix of cells and have tissue formation promotion and cell function regulation [67]. Zhou et al. [68] extracted marine type I collagen from tilapia and prepared electrospun nanofibers with smooth surfaces. In vitro studies have shown that the collagen fibers were conducive to the adsorption of human keratinocytes and significantly promote cell proliferation, with good cell compatibility. However, collagen nanofibers have drawbacks of easy solubility in water, poor thermal stability, and insufficient mechanical properties. Chemical crosslinking treatment is an effective method for improving collagen fibers, but it may be toxic [69]. In addition, other natural polymers such as alginate, gelatin, fibroin, and nucleotides can also serve as good substrates for electrospun fibers and are widely used in wound dressing research [70,71]. However, electrospun nanofibers composed of natural polymers have problems with unstable structures and poor mechanical properties [72].

Synthetic polymers, such as polycaprolactone (PCL), polyvinyl alcohol (PVA), polylactic acid (PLA), polyethylene oxide (PEO) and polyacrylonitrile (PAN), are widely used in electrospun nanofibers because of their excellent mechanical properties, good thermal stability, and processing flexibility [73]. PCL has excellent mechanical properties and controllable biodegradability, which has been approved by the Food and Drug Administration (FDA) for use in many biomedical applications [74]. Due to the lack of functional groups on the surface of PCL [75], surface coating hydrolysis or other modification methods have been proposed, and surface alkali hydrolysis with sodium hydroxide is a simple and effective method [76]. Further, the ammonolysis of PCL has been proven to be beneficial to cell adhesion. Chaiarwut et al. [77] used sodium hydroxide to alkali hydrolyze the electrospun PCL NFM to improve its hydrophilicity and then used carbodiimide to fix the antibacterial peptide Pexiganan on PCL. After treatment, the hydrophilicity of NFM was significantly improved, and the antibacterial rate against gram-negative bacteria could be close to 100%. PVA is a nontoxic and hydrophilic synthetic polymer authorized by the FDA for biomedical and pharmaceutical purposes [78]. However, it has been found that when PVA is used to prepare electrospun nanofibers, the PVA NFM soaked in water will lose its physical integrity and become unstable [79]. Polylactic acid (PLA) has excellent biocompatibility, biodegradability, and eco-friendliness, which is a good medium for drug delivery, tissue engineering and regenerative medicine applications [80]. Compared with conventional medical gauze, electrospun PLA NFMs have good hemocompatibility and wound-healing properties [81,82]. It has been proved that electrospun PLA NFMs are beneficial to the adhesion and migration of skin cells and promote the deposition of collagen [83]. However, PLA has low impact toughness and is sensitive to hydrolysis, which is not conducive to long-term work in the physiological environment [84]. One solution is to obtain PLA stereocomplex by controlling the molecular weight of the homopolymer, which plays a positive role in improving the mechanical properties and hydrolysis resistance of PLA [85]. PAN has good stability and mechanical properties, which have been applied in filtration membranes, aerospace technology and wound dressings [86,87]. Due to the excellent fiber formability of PAN, it is easy to prepare electrospun fibers with good morphology and uniform diameter. It has been found that PAN may have potential antifungal properties [88]. However, PAN nanofibers have the general hydrophobic properties of synthetic fibers. By adding amine groups on the surface of PAN nanofibers through triethylenetetramine or by changing the active nitrile groups on PAN into hydrophilic groups through a chemical reaction, the surface modification of PAN can be attained [89,90]. 

Considering the characteristics of natural polymers and synthetic polymers, their combination can achieve a balance between the mechanical properties and biological functions of electrospun nanofibers. Notably, when natural and synthetic polymers are combined, their physical and chemical properties and their interactions need to be carefully considered. Introducing synthetic polymers mainly enhances the mechanical strength and spinnability of natural polymers. For example, some researchers blended chitosan with PCL for electrospinning to improve the spinnability of chitosan solution and the insufficient mechanical properties of nanofibers [91]. Zulkifli et al. [92] mixed collagen with PVA and hydroxyethyl cellulose for electrospinning, which improved the problems of easy water solubility and insufficient mechanical properties of collagen. Moreover, the composite NFM showed better cell adsorption, growth rate and mobility, which had great potential in skin tissue engineering applications.

### 3.2. Added Functional Factors for Wound Healing

The wound-healing process is dynamic and complex and can be divided into four stages: hemostasis, inflammation, proliferation, and remodeling [93]. The formation of wounds means the beginning of the hemostasis phase, and then platelets, plasma fibers and fibrin form clots to seal the blood flow. During the inflammatory phase, neutrophils, macrophages, and lymphocytes accumulate and are activated, with antimicrobial and apoptotic cell removal effects. The proliferative phase is characterized by the neovascularization and promotion of epithelialization of blood vessels and cells. The remodeling phase is characterized by wound contraction and collagen deposition. The four phases of wound recovery represent the different functional requirements of wound dressings. Some natural polymers have inherent antibacterial or anti-inflammatory functions, but this is insufficient. To strengthen the function of electrospun nanofibers and better promote wound healing, more wound-healing-promoting factors are selectively added into the nanofibers to prepare drug-loaded nanofibers [71,94]. Compared with conventional drug delivery systems, electrospinning technology can give nanofibers faster reaction rates and controllable release rates in the field of drug delivery. Various functions of factors, such as hemostatic [95], anti-inflammatory [96], promote cell proliferation or vascular remodeling and other therapeutic factors [97,98], have been proven to better promote wound healing. Here, wound dressings for hemostasis, antibacterial and wound healing are discussed.

#### 3.2.1. Hemostatic Factors

**Figure 3:**
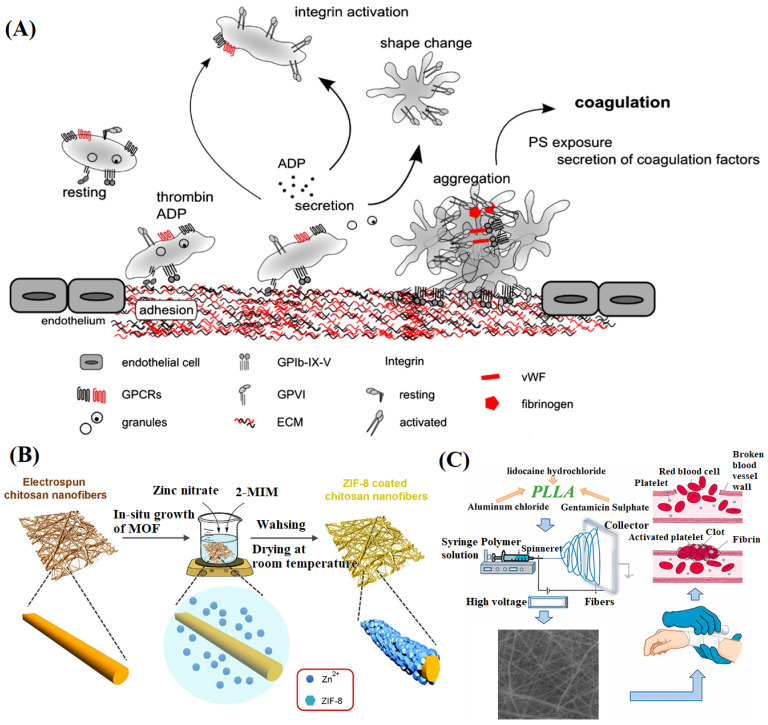
(**A**) Schematic of platelet activation cascade leading to the haemostatic plug formation, reproduced with permission from [99]; (**B**) Schematic representation of in-situ growth of MOF on nanofibrous scaffolds [102]; (**C**) Schematic diagram of electrospun PLLA nanofibrous mats, reproduced with permission from [105].

Rapid hemostasis is the first step in the treatment of wounds. The mechanism of thrombosis is represented in Figure 3A [99]. The traditional method of hemostasis is to use gauze to press the wound to block the blood flow, which has the problems of more blood loss as well as adhesion between the gauze and the wound. To overcome these shortcomings, wound dressings prepared from materials with clotting properties (such as chitosan) or a porous expandable structure or containing hemostatic agents (such as aluminum chloride, tranexamic acid (TXA), and thrombin) have been extensively studied [100]. Wu et al. [101] electrospun composite NFMs by mixing polybutylene succinate and chitosan. The addition of polybutylene succinate effectively improved the spinnability of chitosan. The results showed that when the ratio of chitosan to polybutylene succinate was 9:1, the hemostatic performance of NFM was the best. Lamei et al. [102] introduced tannic acid and zinc-based metal-organic frameworks (MOFs) into electrospun chitosan/PVA blend NFMs (Figure 3B). The results showed that tannic acid could form a synergistic effect with chitosan to stop bleeding in wounds quickly. The zinc-based MOFs endowed fibers with a porous structure conducive to the rapid absorption of blood. Moreover, the presence of zinc ions generated electrostatic interactions with red blood cells, forming a new coagulation pathway. In addition, wound dressings with porous structures can effectively deal with pathogenic bleeding. Gu et al. [103] conducted ultrasonic treatment on electrospun chitosan NFMs to obtain a porous structure. After ultrasonic treatment, the porosity of chitosan NFM could be increased by about 20%, and the water absorption time could be reduced by nearly 100 s. Compared with commercial hemostatic gauze, the porous chitosan NFM was 1.35 times more effective in clotting blood. 

Aluminium chloride is a widely used material to stop bleeding [104]. Nasser et al. [105] electrospun poly-l-lactic acid (PLLA) NFM containing aluminum chloride by blending method (Figure 3C). The results showed that aluminum chloride with 33% *w*/*w* had the best hemostatic performance. The NFM had a shorter blood clotting time and a stronger blood absorption capacity than traditional bandages. In another study, kaolin was added to the electrospun chitosan/PEO blend fibers. The layered structure and micro-pores on the surface of kaolin absorbed water in the blood and accelerated the aggregation of platelets and thrombin to achieve rapid hemostasis [106]. Sasmal et al. [107] introduced TXA into the electrospun chitosan/PVA NFM and evaluated its release and hemostatic effect. The results showed that TXA was released 90% within 10 h, and the presence of TXA reduced the blood clotting time by stabilizing coagulation. Mendes et al. [108] implemented thrombin loading on PEO nanofibers through electrospinning. Studies on wound healing in vitro and in vivo showed that thrombin was released by water at the wound site as the NFM degraded, accelerating the clotting process. Moreover, the NFM was suitable for wounds with different morphologies and could be removed without external force after application.

#### 3.2.2. Antibacterial Factors

Wound infection is a common problem in clinical practice that can not only affect the normal process of wound recovery but also aggravate the pain of patients and even endanger their lives. To effectively reduce the probability of wound infection, antimicrobial agents are added to wound dressings, which commonly include antibiotics, antimicrobial peptides, metals, and metal oxides [109].

**Figure 4:**
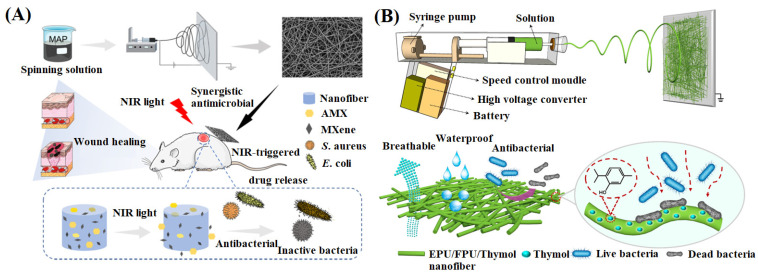
(**A**) Schematic diagram of the preparation of antibacterial nanofiber membrane containing AMX, reproduced with permission from [111]; (**B**) Schematic diagram of in-situ electrospun equipment, reproduced with permission from [112].

Antibiotics specifically affect inflammation caused by wound infection, but inappropriate dosage can lead to allergies and bacterial resistance [110]. Xu et al. [111] added amoxicillin (AMX) and MXene into a PVA spinning solution and prepared an antibacterial composite NFM (Figure 4A). The release rate of AMX could be controlled by PVA, and MXene, as a photothermal agent, could convert near-infrared light into heat for local hyperthermia of the wound surface, thus promoting the release of AMX and assisting in sterilization by destroying the bacterial membrane. In a mouse skin defect model, the NFM showed outstanding bacteriostatic effects and wound healing ability after treating *Staphylococcus aureus* infection. Yue et al. [112] used fluorinated polyurethane and ethanol-soluble polyurethane to prepare a waterproof and breathable NFM through in-situ electrospinning technology, which could protect the wound from external stimulation (Figure 4B).

Further, thymol was added to the NFM, which made the drug-loaded NFM have good antibacterial effects against *Escherichia coli* and *Staphylococcus aureus*. Sun et al. [113] prepared puerarin-loaded electrospun composite NFMs with silk protein and polyvinylpyrrolidone (PVP) as matrix materials. It has been proven that puerarin improved the porosity, hydrophilicity, and antioxidant capacity of NFMs. In in vivo studies, the composite NFMs reduced the inflammatory response, promoted cell adhesion and proliferation, and accelerated wound healing. 

Antimicrobial peptides are a new antibacterial agent with little drug resistance, strong bactericidal ability, good thermal stability, and no immunogenicity. Yu et al. [114] used chitosan and PEO as matrices, added different contents of antibacterial peptides, and prepared antibacterial nanofibers using electrospinning technology. This NFM had a good inhibitory effect on *Escherichia coli* and *Staphylococcus aureus*. In the animal wound healing experiment, the wound healing rate of NFM containing antimicrobial peptides was better than that of NFM without drug loading and common gauze. Metals and metal oxides are widely used as antibacterial materials in wound dressings, among which ZnO quantum dots are a low-toxicity and inexpensive nanomaterial. Li et al. [115] prepared PCL/collagen porous scaffolds containing ZnO quantum dots, which showed that adding ZnO quantum dots endowed the porous scaffold with high antibacterial performance against *Staphylococcus aureus* and *Escherichia coli*. Meanwhile, the composite scaffold exhibited excellent cell compatibility in promoting cell proliferation. In addition, the composite scaffold with vascular endothelial growth factor was proven to accelerate wound healing by promoting the expression of transforming growth factor-β (TGF-β) and vascular factor in tissues during the early stages of wound healing.

#### 3.2.3. Growth Factors

The four stages of wound healing involve different cells, growth factors and proteins. For example, activated platelets at the hemostatic stage can secrete a large number of growth factors, such as transforming growth factors (TGF-α, TGF-β) and platelet-derived growth factors, which promote the migration of inflammatory cells [116]. Therefore, the introduction of growth factors is very attractive for wound healing. Here, we mainly discuss the effects of introducing growth factors on wound recovery. 

Skin reconstruction is accompanied by the release of growth factors [117], which promote cell proliferation and granulation tissue formation and play an important role in different stages of wound healing [118,119]. For example, epidermal growth factor (EGF) can promote the proliferation and epithelialization of keratinocytes and has synergistic effects with fibroblast growth factor. Platelet-derived growth factor (PDGF) can facilitate fibroblast proliferation and granulation tissue growth, playing a role in the initial stage of wound healing. Vascular endothelial growth factor (VEGF) accelerates angiogenesis and granulation tissue formation. Fibroblast growth factor (FGF) promotes mitosis and angiogenesis and plays a role in the later stage of wound recovery, among which basic FGF (bFGF) facilitates cell proliferation, migration, and differentiation. However, the stability of growth factors is poor, and their half-lives are short. The introduction of growth factors requires consideration of their concentration and biological activity. Electrospun nanofibers with a similar skin structure are undoubtedly a good growth factor carrier, which can provide a controlled release of therapeutic factors and protect their biological activity.

**Figure 5:**
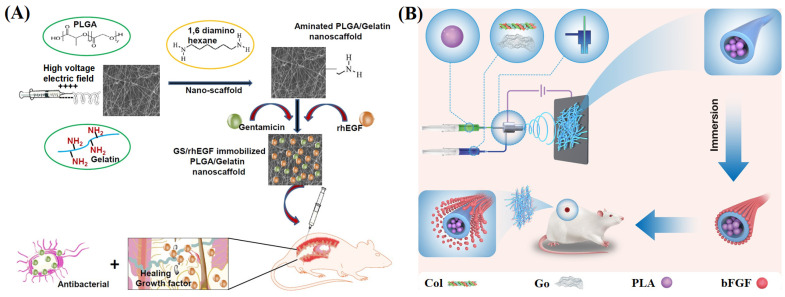
(**A**) Process of fabrication of aminolyzed PLGA/Gelatin nanoscaffolds and subsequent application on diabetic wounds, reproduced with permission from [120]; (**B**) Col-GO and PLA through coaxial electrospinning to form core–shell fiber scaffolds for skin tissue engineering applications, reproduced with permission from [121].

Dwivedi et al. [120] prepared electrospun blended NFMs with poly(d, l-lactide co glycolide) (PLGA)/gelatin as the matrix. They introduced recombinant human epidermal growth factor (rhEGF) and gentamicin sulfate on their surface to accelerate the treatment of diabetes wounds (Figure 5A). The composite NFM retained the stability and bactericidal properties of gentamicin sulfate, releasing only 36.64 ± 0.51% within 12 h, and the maximum inhibition rate of bacterial growth could reach 98.73 ± 0.68%. In the wound healing model of a mouse, the NFMs containing rhEGF played a positive role in the initial stage of wound healing, significantly increasing the wound closure rate. Chen et al. [121] prepared collagen/GO nanofiber membranes containing bFGF through electrospinning (Figure 5B). The maximum cumulative release rate of bFGF in the NFM containing bFGF was 30.94 ± 7.77%, with a release time of up to 27 days. In the wound healing model, the NFM showed a 96.39 ± 0.66% wound healing rate, and the promoting effect of growth factors on wound healing was demonstrated. Taborska et al. [122] used poly(L-lactide-co-ε-caprolactone)/PCL nanofibers as a matrix containing human platelet lysates (hPL), and the fibrin network of VEGF and FGF as a coating to prepare a composite wound dressing. The results showed that the fibrin network was a good receptacle for bioactive molecules, and the sustained release of growth factors and hPL from the coating significantly increased the survival rate of human saphenous vein endothelial cells in collagen wound models.

#### 3.2.4. Other Therapeutic Factors

**Figure 6:**
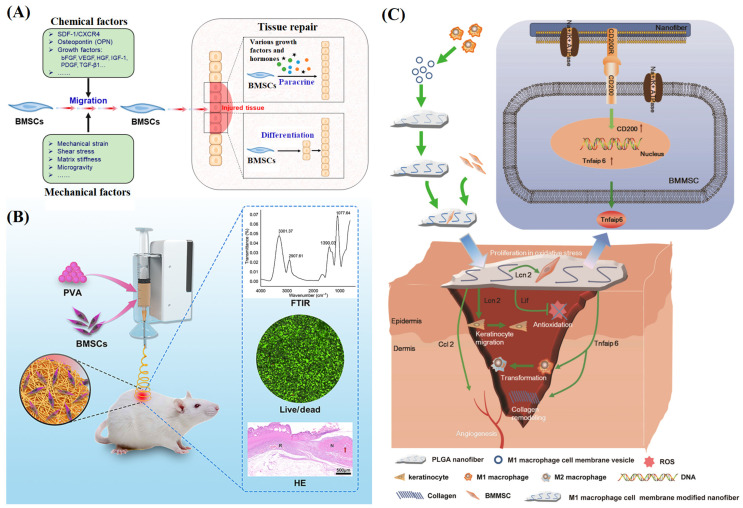
(**A**) Proposed schematic diagram of the relationship between BMSC migration and tissue repair, reproduced with permission from [124]; (**B**) Schematic diagram of preparation of polymer fibers for wound dressing by in situ cell electrospun using a portable handheld electrospinning apparatus, N represented normal tissue and R represented repaired tissue, the red arrow represented appendages, reproduced with permission from [125]; (**C**) LPS/IFN-γ activated RAW264.7 cell (M1-like murine macrophage cell) membrane modified PLGA nanofibers with BMMSCs attachment promote diabetic wound healing, reproduced with permission from [126].

Cell therapy is a treatment that uses living cells to renew and regenerate damaged tissue. Pluripotent cells such as macrophages, endothelial progenitors, and stem cells have been used in cell therapy [123]. Among them, stem cells can self-renew and differentiate into various cells, which is of great significance for the repair and reconstruction of damaged tissues and shows great potential in wound healing. Bone marrow mesenchymal stem cells (BMSCs) are useful in the treatment of different types of wounds (Figure 6A) [124]. Xu et al. [125] prepared a PVA/BMSCs NFM through a handheld electrospinning device (Figure 6B). The good biocompatibility of NFM was verified by cytotoxicity and cell proliferation experiments. The introduction of BMSCs had a positive effect on the formation of granulation tissue and epithelialization in full-layer skin wounds of rats. Compared with blank control, BMSCs could significantly accelerate wound healing. In another study, a PLGA electrospun NFM with LPS/IFN-gamma activated macrophage cell membrane was constructed and loaded with BMSCs (Figure 6C). In vitro oxidative stress tests, the modified NFM had been shown to promote BMSCs proliferation and keratinocyte migration. In a diabetic wound healing model, the composite NFM exhibited faster-epithelialized regeneration, collagen remodeling and angiogenesis, accelerating wound healing, compared with the fibrous membrane without modification of the cell membrane [126].

The combination of stem cells and growth factors is an effective strategy to enhance wound healing. Fu et al. [127] constructed a composite sponge material loaded with nano-adipose tissue by using electrospun short fibers modified by polydopamine, in which nano-adipose tissue contained a variety of cells such as adipose-derived stem cells and could secrete various growth factors such as VEGF. The composite dressing could promote angiogenesis by releasing cells and growth factors, accelerate the growth of granulation tissue, and close the wound through granulation tissue, providing an enabling environment for tissue regeneration and repair. In addition, it is worth noting that the addition of therapeutic factors needs to consider their compatibility with the hydrophilic properties of polymers. 

By designing the components of electrospun nanofibers, different parts of the wound healing process can be well promoted. The applications of electrospun nanofibers in hemostasis, antibacterial, and wound healing promotion have been summarized in Table 2.

## 4. Structural Design

### 4.1. Structural Design of Single Nanofiber

#### 4.1.1. Porous Structure

**Figure 7:**
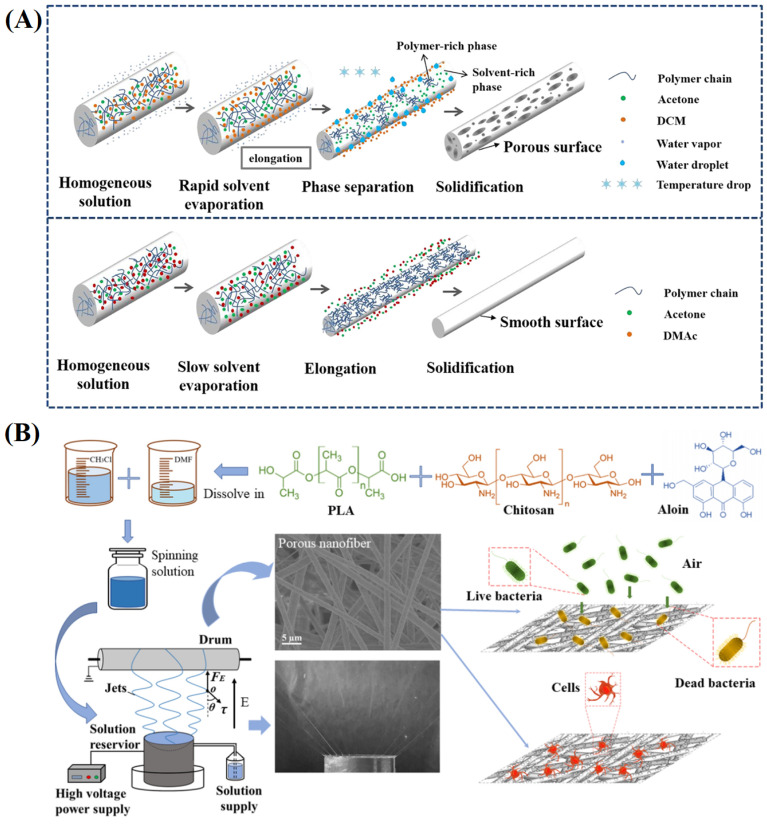
(**A**) Schematic diagrams of the formation of porous fibers and nonporous fibers, reproduced with permission from [131]; (**B**) Fabrication process of porous PLA/CS NFMs and subsequent applications, reproduced with permission from [135].

Porous nanofibers have a larger surface area, rich internal space and surface-active sites, which provide a good platform for drug delivery and osmotic absorption and accelerate the diffusion, transmission, or transformation of substances. The preparation methods of porous nanofibers mainly include post-treatment and phase separation. Post-treatment refers to the selective removal of a component from blended nanofibers to form pores [128]. Phase separation is to form porous structures by adjusting spinning parameters to take advantage of the space occupied by the volatilisation or evaporation of the liquid phase during the fiber forming process, with the temperature difference being the driving force behind the phase separation [129,130]. Chen et al. [131] used a phase separation method to prepare porous cellulose acetate nanofibers containing thymol (Figure 7A). The principle was that after the jet was ejected from the spinneret, the rapid volatilization of highly volatile solvents would decrease the fiber surface temperature, which could induce the phase separation of the jet at low temperatures.

Moreover, there were water droplets liquefied by water vapor and concentrated solvents on the fiber surface. During the drying stage, the space occupied by the solvents and water droplets formed pores. Compared with drug-loaded nonporous nanofibers, drug-loaded porous nanofibers have a slower initial release rate, longer drug release time and higher drug utilization, which can improve the proliferation of cells, exhibiting better cell compatibility [132]. The effect of pores in nanofibers on the interaction between living cells and nanofibers has been confirmed, which is beneficial for wound dressings’ biocompatibility and antibacterial efficacy [133]. Lanno et al. [134] electrospun porous PCL nanofibers under high relative humidity. Compared to nonporous PCL nanofibers, porous PCL nanofibers were more conducive to the adsorption and growth of fibroblasts. Yin et al. [135] adopted free surface electrospinning technology to achieve the batch preparation of drug-loaded porous PLA/CS nanofibers (Figure 7B), which had good swelling properties, excellent blood coagulability, and biocompatibility.

#### 4.1.2. Beaded Structure

**Figure 8:**
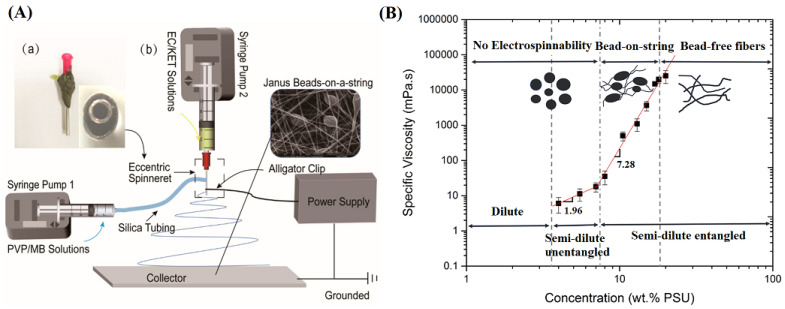
(**A**) The implementation process of side-by-side electrospinning: (**a**) an image of a home-made eccentric spinneret; (**b**) schematic drawing of the home-made electrospinning system, reproduced with permission from [138]; (**B**) Plot of specific viscosity versus concentration for solutions, reproduced with permission from [141].

Beaded nanofibers have been proven to be beneficial for some emerging applications, including air filtration [136], superhydrophobic surface modification [137] and drug delivery (Figure 8A) [138,139]. The formation of beads is mainly affected by the concentration, electrical conductivity, and surface tension of the spinning solution. Higher concentration and higher electrical conductivity are conducive to the formation of beadless fibers. In contrast, higher surface tension tends to form beaded fibers because the formation of beads is beneficial to reducing the surface energy of the fluid. Li et al. [140] electrospun beaded nanofibers with controllable diameter by adjusting the concentration of PLGA and the ratio of solvent. The results showed that within a certain concentration range, with the increase of PLGA concentration, the diameter of beads increased, and the shape of beads changed from elliptical to elongated. Rasouli et al. [141] explored the effects of spinning solution concentration, voltage, and flow rate on the morphology of beaded polysulfone nanofibers (Figure 8B), which indicated that when the concentration of polysulfone was 7–18 wt%, the fibers exhibited a bead-like morphology. When the concentration was constant, the number of beads decreased as the flow rate or voltage increased. Moreover, as the voltage increased, the bead shape changed from spherical to spindle.

The bead structure of nanofibers has a positive effect on drug delivery, which can increase the embedding depth of the drug by coating the drug in the beads with a larger diameter, thus alleviating the initial burst drug release and achieving a longer-term sustained drug release [142,143]. Saeed et al. [144] prepared curcumin-loaded beaded nanofibers using PCL and PVA. The presence of curcumin and PVA increased the antibacterial and osmotic absorption of the dressing. In drug release experiments, increasing the number of beads effectively reduced the sudden release of curcumin. In addition, the wound dressing obtained through the beaded nanofibers had excellent osmotic absorption ability and biocompatibility, and the bactericidal rate could reach 100% when containing 5% curcumin.

#### 4.1.3. Core-Shell Structure

Core-shell nanofibers are generally obtained by coaxial electrospinning equipment, which can reduce the requirement for spinning solution and effectively improve the drug burst in drug-loaded fibers. Meanwhile, unstable proteins and other biological factors can be wrapped in the core layer of core-shell nanofibers by coaxial electrospinning, which reduces the interaction between organic polymers and water-based biological molecules and better protects the biological activity of biological molecules [145]. However, in the preparation of core-shell nanofibers, the selection of polymers and solvents, as well as the flow rates of shell and core layer solutions, should be considered, which can affect the integrity of the core-shell structure and the diameter of core-shell nanofibers [146,147].

**Figure 9:**
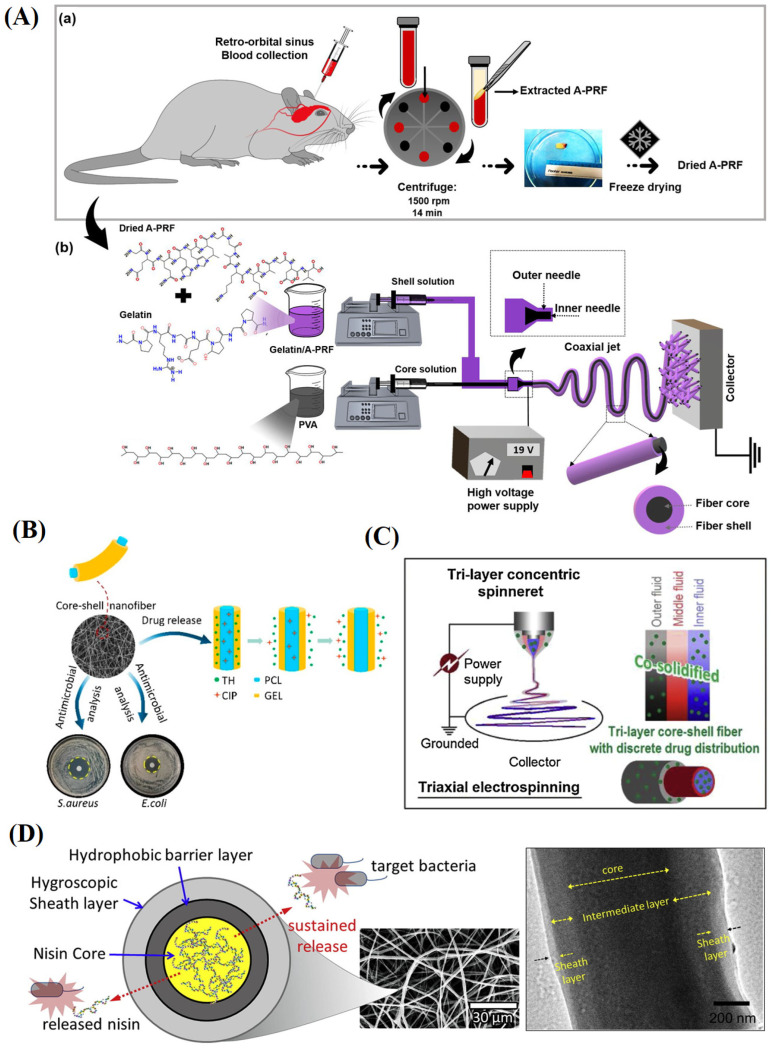
(**A**) (a) Extraction and preparation of dried A-PRF. (b) Preparation of core-shell nanofibers by coaxial electrospinning method, reproduced with permission from [148]; (**B**) The preparation of coaxial nanofibers with co-delivering of CIP and TH antibiotics, reproduced with permission from [149]; (**C**) Diagram of modified triaxial electrospinning for creating tri-layer core-shell nanofibers with discrete drug distributions, reproduced with permission from [151]; (**D**) Schematic diagram of the preparation of electrospun triaxial fiber membranes, the black arrow represented fiber diameter, reproduced with permission from [152].

Tavakoli et al. [148] prepared composite nanofibers with core-shell structures by coaxial electrospinning technology using PVA as the core spinning solution as well as gelatin and advanced platelet-rich fibrin mixture as the shell-spinning solution (Figure 9A). The core-shell nanofibers showed a high specific surface area, porosity, and hydrophilicity. Compared with blended nanofibers, core-shell nanofibers had better mechanical properties, higher cell proliferation and adhesion rates, and accelerated wound healing. The characteristics of the core-shell structure also allow the loading of the dual-drug system. Lin et al. [149] introduced the hydrophobic ciprofloxacin into PCL as the core layer and the hydrophilic tetracycline hydrochloride into gelatin as the shell layer using coaxial electrospinning. They developed an antibacterial wound dressing that delivered two antibiotics (Figure 9B). The results showed that the core-shell nanofibers had strong antibacterial activity against *Escherichia coli* and *Staphylococcus aureus and* good biocompatibility.

Furthermore, hollow nanofibers can be prepared using soluble or volatile solutions as the core spinning solution. Yilmaz et al. [150] prepared hollow PLA/polyurethane nanofibers using a mixed solution of PLA and polyurethane as the shell-spinning solution and a PVP solution as the core spinning solution. Compared to solid PLA/polyurethane nanofibers, hollow-structured nanofibers had smaller diameters, higher tensile strength, and liquid absorption capacity.

#### 4.1.4. Multicore-Shell Structure

Multicore-shell or multi-layer nanofibers have also been extensively studied and produced by multiaxial electrospinning. Yang et al. [151] used triaxial electrospinning to prepare three-layer nanofibers with the drug model ketoprofen in the inner and outer layers and pure cellulose acetate in the middle layer (Figure 9C). Compared with core-shell nanofibers, the system had a better performance in both the drug’s initial and sustained release phases. Similarly, Han et al. [152] designed three-layer nanofibers with an outer layer of hygroscopic cellulose acetate, an inner layer of nisin, and a hydrophobic middle layer of PCL to prevent the sudden release of drugs in the inner layer (Figure 9D). The antibacterial activity test showed that the bactericidal rate of nisin was more than 99.99% within five days and the nanofibers provided more sustained antimicrobial activity compared with coaxial and single homogenous nanofibers. Nagiah et al. [153] presented a new type of three-layer nanofibers with PCL as the inner layer, gelatin as the middle layer and PLGA as the outer layer, which had significantly better mechanical properties than pure PLGA and gelatin (core)/PLGA (shell) nanofibers. The drug release tests also showed their excellent drug sustained release. The unique structure of multicore-shell nanofibers has great potential in drug delivery and wound dressings. However, the selection of materials presents a challenge for preparing multicore-shell nanofibers. 

#### 4.1.5. Self-Assembled Multi-Layer Structure

**Figure 10:**
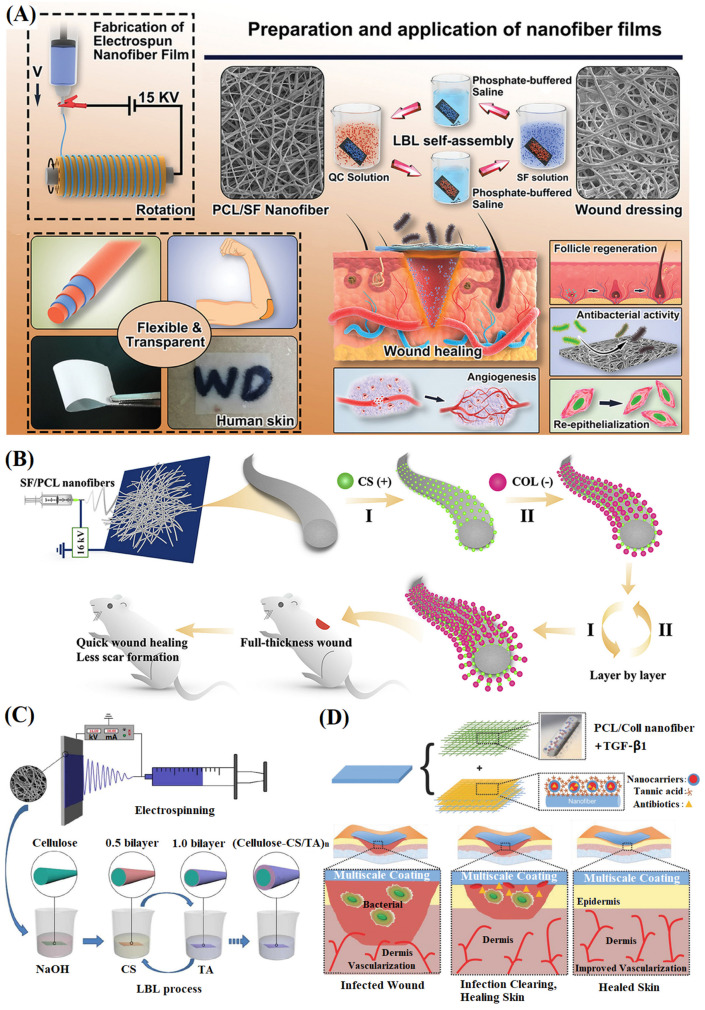
(**A**) General idea of highly flexible and broad antibacterial nanodressing, reproduced with permission from [154]; (**B**) Schematic diagram illustrating LBL modified nanofibrous mats (I for step 1, II for step 2), reproduced with permission from [156]; (**C**) Schematic diagram illustrating the LBL modification on nanofibrous mats, reproduced with permission from [157]; (**D**) Schematic representation of a modular system to simultaneously stimulate wound healing and mitigate infection, and BCM/TA coatings enhance NHDFs migration rate and proliferation on PCL/Coll NFs, reproduced with permission from [160].

Layer-by-layer self-assembly technology is a common method to prepare multi-layer structured nanofibers by alternating deposition of substrates through electrostatic interactions between electrolytes, which can be used to improve the performance of electrospun nanofibers. Hu et al. [154] deposited positively charged quaternary ammonium salt chitin and negatively charged silk fibroin layer by layer on the surface of electrospun PCL nanofibers to prepare multi-layer nanofibers through self-assembly technology (Figure 10A), which has stronger antibacterial properties, angiogenesis, and collagen deposition. To increase the anti-inflammatory and reactive oxygen elimination functions of the dressing, in the follow-up study, the research team interactively deposited dihydromyricetin and quaternized chitosan on the electrospun PCL nanofibers and characterized their biocompatibility and wound application. The results showed good hydrophilicity and biocompatibility, which could effectively stop bleeding, resist bacteria, diminish inflammation, remove reactive oxygen species, promote cell migration, and facilitate wound healing [155]. This provides an important reference value for the preparation of wound dressings with multi-component and multi-layer structural designs. Wu et al. [156] deposited positively charged chitosan and negatively charged collagen on electrospun silk fibroin/PCL nanofibers through layer-by-layer self-assembly technology (Figure 10B). The deposition of chitosan and collagen increased the diameter of nanofibers, resulting in irregular protrusions on their surface, and significantly improved the mechanical properties and hydrophilicity of nanofibers, exhibiting excellent antibacterial activity. The rat model test showed that the multi-layer nanofibers could accelerate wound healing, promote collagen deposition, and reduce scar formation through the TGF-b/Smad signaling pathway, which had great potential in the application of skin regeneration. Huang et al. [157] deposited positively charged chitosan and negatively charged tannic acid on the alkali hydrolyzed cellulose acetate nanofibers to prepare multi-layer nanofibers (Figure 10C). The results showed that the layer-by-layer self-assembly technology could effectively improve nanofibers’ hydrophilicity and mechanical properties, and the multi-layer nanofibers had significant antibacterial activity against *Escherichia coli* and *Staphylococcus aureus*.

Furthermore, the effect of the number of coating layers on the properties of multi-layer nanofibers has been investigated. Huang et al. [158] used electrospun cellulose acetate/PCL composite nanofibers as the substrate. They applied layer-by-layer self-assembly technology to repeatedly deposit positively charged chitosan and negatively charged type I collagen. With the increase in the number of coating layers, the fiber morphology became rough and irregular bulges appeared, which might be caused by uneven deposition. When the number of layers was 10, the mechanical properties of nanofibers increased significantly, but when the number of layers was 15 and above, the mechanical properties of nanofibers decreased significantly. When the number of layers was 20, some filamentous films appeared between adjacent fibers, restoring the mechanical properties of nanofibers to the level of the uncoated sample. In addition, the greater the number of coating layers, the fewer apoptotic cells on the NFM, and the higher the biocompatibility and support for cell proliferation of the NFM. Similarly, Tu et al. [159] deposited carboxymethyl chitosan on the electrospun silk fibroin nanofibers’ surface by layer self-assembly treatment to prepare multi-layer nanofibers. It was found that with the increase of carboxymethyl chitosan layers, nanofibers’ mechanical properties, biocompatibility, and antibacterial ability were significantly improved, and the effect of inhibiting bacteria would be better.

Biocompatible micelles can also be coated on degradable nanofibers through layer-by-layer self-assembly technology to build a dual-release system. Albright et al. [160] first introduced transforming growth factor-β1 into electrospun PCL/collagen nanofibers and then deposited biocompatible nanomicelles based on polypeptide block polymer and tannic acid on the surface of nanofibers to form multi-layer nanofibers (Figure 10D). Transforming growth factor-β1 could attract inflammatory cells, promote angiogenesis, and stimulate the differentiation of myofibroblasts, and biocompatible micelles could prevent wound infection. Compared with the uncoated nanofibers, the coated nanofibers had considerable fibroblast adhesion and diffusion ability and significantly enhanced fibroblast migration. The influence of electrospun nanofiber structures on wound healing has been summarized in Table 3.

### 4.2. Structural Design of NFMs

#### 4.2.1. Ordered Structure

**Figure 11:**
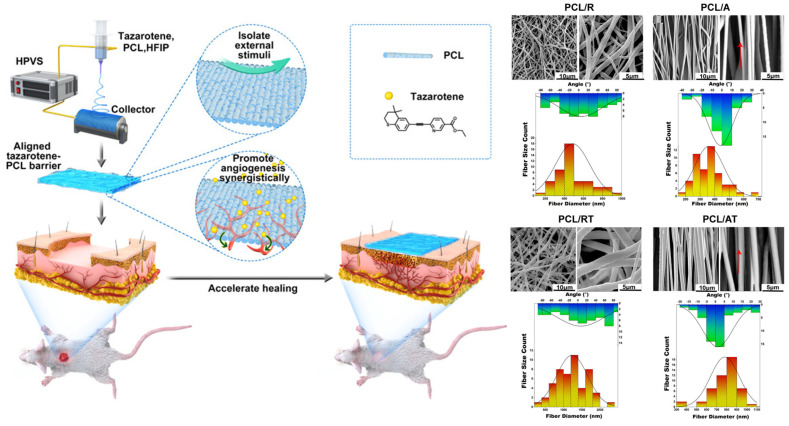
Preparation of Aligned Electrospun Membrane and SEM micrographs of each group. fibers’ direction, as shown by the red arrows. Membranes with histograms for the diameter (red) and orientation (blue) distributions of the nanofibers from corresponding SEM images, reproduced with permission from [165].

The nanofibers collected through a flat plate collector are generally randomly oriented, caused by the irregular movement of charged jets. However, randomly arranged NFMs have relatively poor mechanical properties, and increasing the orientation of nanofibers is an effective strategy for improving the mechanical properties of NFMs [161,162]. Meanwhile, the influence of nanofiber arrangement on cell migration and phenotypic expression has also been widely reported [163,164], which shows great potential in wound healing. To obtain electrospun nanofibers with high orientation, NFMs with ordered structures are prepared by introducing additional mechanical force fields, and electric or magnetic fields to control the trajectory of charged jets. Moreover, collecting electrospun nanofibers using a rotary collector is a common method for preparing ordered NFMs. Zhu et al. [165] used a high-speed rotating (3000 rpm) collector wrapped in aluminum foil to collect ordered NFMs and prepared PCL NFMs loaded with tazarotene(Figure 11). Biocompatibility testing showed that the ordered NFMs promoted cells to creep along the main fiber direction, forming elongated cells, which was conducive to the growth of cell populations towards a unified direction. The promoting effect of ordered NFMs on angiogenesis had also been demonstrated in the Matrigel experiment.

**Figure 12:**
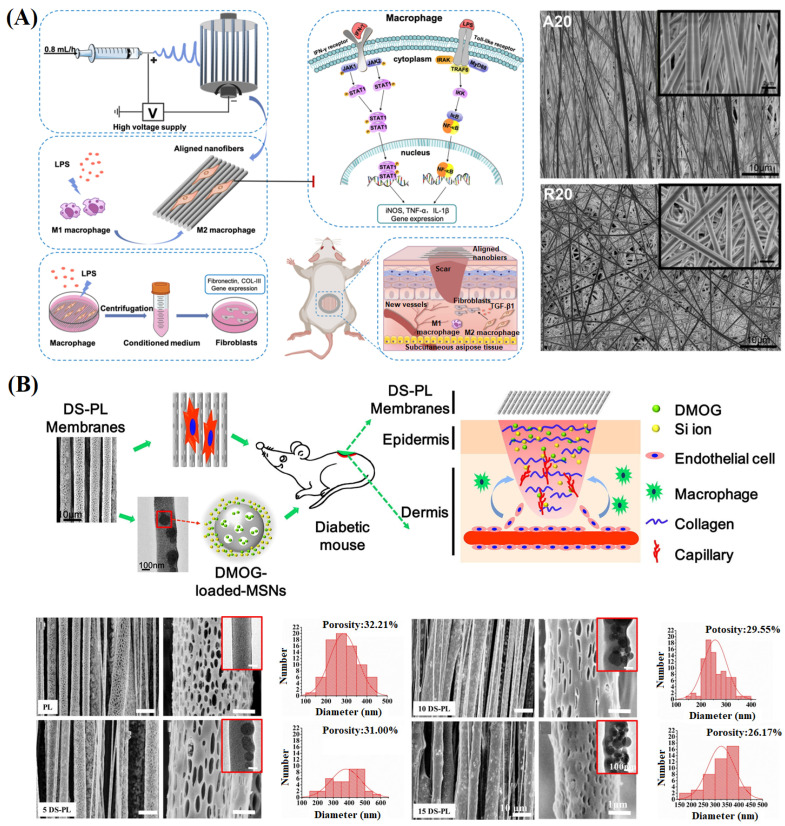
(**A**) The fabrication of aligned electrospun PLLA nanofibers and their use in wound healing, and characterization of electrospun membranes: SEM images, stress–strain curves, elasticity modulus and water contact angles of the aligned and random electrospun membranes, reproduced with permission from [166]; (**B**) Preparation of an aligned porous electrospun fibrous membrane with controlled drug delivery, and SEM and TEM images of the aligned porous electrospun membranes and the corresponding pore size distribution of them (the corresponding TEM image inserted in each SEM image showed the DS particles incorporated within the nanofibers), reproduced with permission from [167].

Xie et al. [166] studied the effect of aligned electrospun nanofibers on macrophage polarization in a lipopolysaccharide-induced inflammatory environment (Figure 12A). The results showed that aligned nanofibers could down-regulate the pro-inflammatory M1 phenotype, up-regulate the pro-healing M2 phenotype and inhibit M1 macrophage polarization via the JAK-STAT and NF-KB signaling pathways. In animal experiments, aligned nanofibers could alleviate mouse wound inflammation, promote angiogenesis, and accelerate wound healing by conditioning the macrophage phenotype, indicating that the structural design of NFMs could provide a new strategy for their application in macrophage polarization and wound healing. Furthermore, Ren et al. [167] prepared ordered porous PLLA NFMs containing dimethyloxalylglycine (DMOG)-loaded mesoporous silica nanoparticles (DS) (Figure 12B). The results showed that aligned fibers had significant impacts on directing the cellular alignment and migration, and finally promoted angiogenesis. The porous structure increased the specific surface area of fibers and provided more sites for cell adhesion. The ordered porous NFMs with DScould promote cell attachment, migration, and revascularization and significantly promote collagen deposition in diabetes wounds.

#### 4.2.2. Asymmetric Double-Layer Structure

To simulate the structure and characteristics of human natural skin epidermis and dermis and improve the therapeutic effect of wound dressings, asymmetric double-layer dressings have been developed. Generally, asymmetric biomimetic dressings comprise a tight outer layer and a porous inner layer, which can effectively prevent bacterial penetration and wound drying. In addition, 3D printing has gained widespread popularity due to its ability to precisely control the sample aperture and obtain the desired 3D morphology of samples. However, most 3D printing lacks the simulation of extracellular matrix. The combination of 3D printing and electrospinning technology can simultaneously realize the controllable construction of 3D morphology and simulation of extracellular matrix, which can be divided into three categories. One is to use short nanofibers as components of 3D printing ink [168], the other is to use 3D printing to improve the stability of electrospun jet [169], and the other is to combine 3D printing samples with electrospun NFMs to simulate the gradient structure of the skin surface and dermis [170].

**Figure 13:**
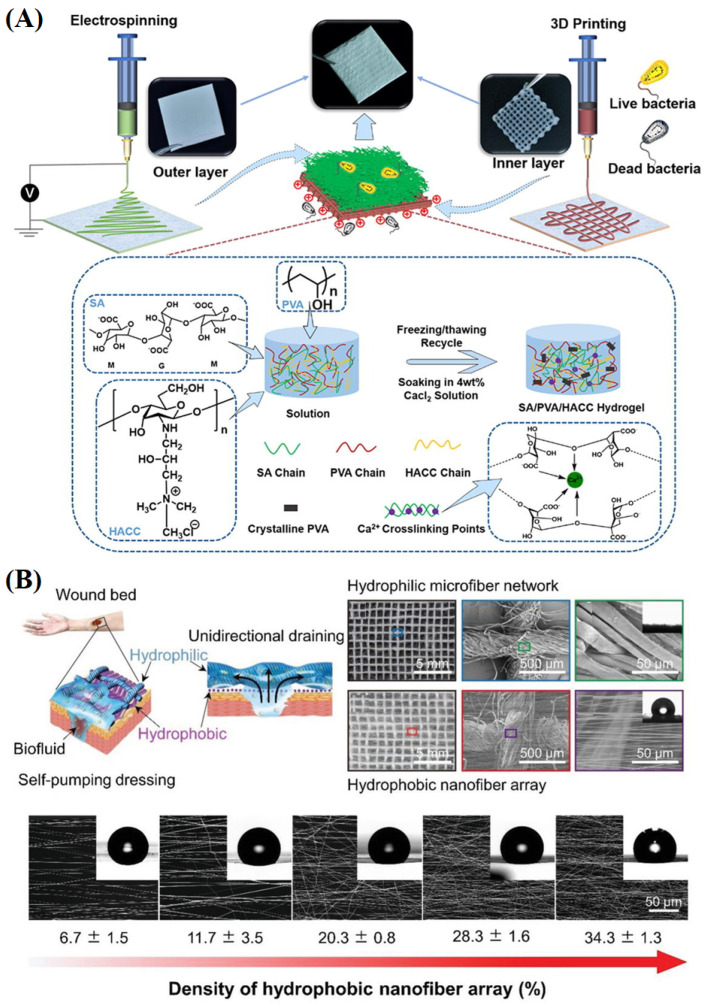
(**A**) Schematic diagram of the double-layer asymmetric dressing through electrostatic spinning and 3D printing, reproduced with permission from [172]; (**B**) Proposed design of the self-pumping dressing for wound healing promotion by draining excessive biofluid and demonstration of unidirectional fluid draining capability of the self-pumping dressing by comprising with the conventional dressing, reproduced with permission from [173].

Liu et al. [171] used electrospinning to prepare a hydrophobic PCL NFM as the outer layer to simulate the density and permeability of the epidermis. They applied low-temperature 3D printing technology to fabricate the hydrophilic inner layer of the dressing with chitosan and copper-doped Laponite, which was responsible for killing bacteria and promoting wound healing. The results showed that the asymmetric dressing had good biocompatibility and antibacterial properties and significantly promoted the migration of endothelial cells, which had a positive impact on wound healing. Zhang et al. [172] prepared an electrospun PCL/PLA composite NFM as the hydrophobic outer layer. They fabricated a porous hydrogel scaffold with sodium alginate, chitosan and PVA through 3D printing as the hydrophilic inner layer of dressing, thus obtaining a double-layer bionic composite membrane (Figure 13A). The results indicated that the bionic skin dressing had high hydrophilicity, porosity, and mechanical properties, which could effectively inhibit the growth of *Staphylococcus aureus* and promote cell proliferation while absorbing excess tissue osmotic fluid to keep the wound moist.

Interestingly, Shi et al. [173] designed a wound dressing with self-pumping absorption capacity based on electrospinning technology (Figure 13B). The wound dressing could remove excess osmotic fluid from the wound in one direction by covering a hydrophilic NFM on a hydrophobic NFM. In the wound healing test of the mouse back, the self-pump dressing showed faster healing speed. 

#### 4.2.3. Multi-Layer Structure

**Figure 14:**
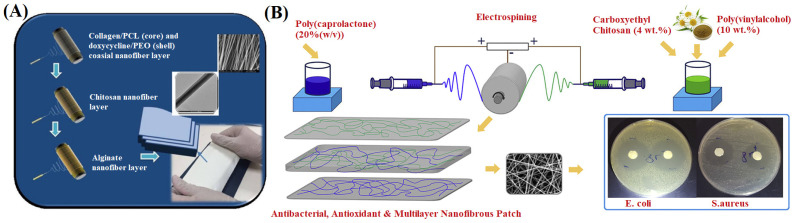
(**A**) Schematic diagram of the preparation of three-layered doxycycline-collagen loaded nanofiber, reproduced with permission from [177]; (**B**) Schematic diagram illustrating multilayer nanofibrous patch comprising chamomile loaded carboxyethyl chitosan/poly(vinyl alcohol) and polycaprolactone, reproduced with permission from [178].

A multi-layer NFM can be obtained by folding or stacking electrospun NFMs, which can promote the agglutination of platelets and blood cells, help to rapidly form a fibrillar protein reticulum on the wound surface, and complete hemostasis due to its high expansion rate [174]. Leung et al. [175] stacked 40 layers of electrospun NFMs, then pressed and sintered them to prepare stable multi-layer NFMs. Song et al. [176] first seeded human fetal osteoblasts on both sides of the electrospun PCL/apatite NFMs and then stacked the NFMs loaded with cells layer by layer in the nanofiber box obtained from origami, thus obtaining a 3D multi-layer wound dressing loaded with cells. Furthermore, multi-layer NFMs can also be obtained by layer-by-layer spinning. Tort et al. [177] obtained a multi-layer wound dressing composed of sodium alginate NFM, chitosan NFM and PCL/collagen core-shell NFM by layer-by-layer electrospinning method (Figure 14A). The sodium alginate and chitosan NFMs as the inner layer could promote contact between the dressing and the wound and shorten the inflammatory period. The PCL/collagen core-shell NFM containing doxycycline provided mechanical support for cell migration and wound remodeling, effectively promoting wound healing. Shokrollahi et al. [178] prepared a three-layer composite NFM by electrospinning, using chamomile, carboxy chitosan and PVA as the inner layer materials, PCL as the outer layer materials, and inner and outer layer materials mixed as the middle layer material (Figure 14B). The inner layer containing hydrophilic chamomile could establish a good interface with the wound, the outer layer of hydrophobic PCL provided strength for the dressing, and the middle layer was a cohesive promoter between the hydrophilic and hydrophobic layers, making the three-layer composite NFM have good mechanical properties, high bacteriostatic effects, and good biocompatibility.

**Figure 15:**
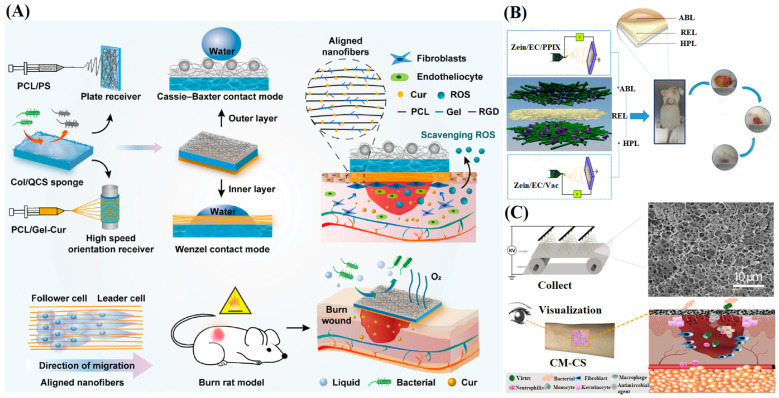
(**A**) Schematic of the biomimetic asymmetric composite dressing preparation and Morphology and wettability characterization of the asymmetric composite dressing, reproduced with permission from [179]; (**B**) Schematic illustration of the preparation of multilayer composite (MC) membrane by electrospinning. ABL, antibacterial layer; RFL, reinforcement layer; HPL, healing promotion layer; BC, bacterial cellulose, reproduced with permission from [180]; (**C**) Schematic illustration of the preparation of CM-CS and Schematic diagram of CM-CS covering the skin wound, reproduced with permission from [181].

Sponges not only have excellent hydrophilicity and swelling rat, but also provide support and antibacterial functions, so they can also participate in the formation of multi-layer wound dressings. He et al. [179] first prepared a collagen/quaternary ammonium chitosan sponge by the freeze-drying method. Then, they prepared the superhydrophobic outer layer of PCL/polystyrene microspheres and the hydrophilic inner layer of PCL/gelatin composite NFMs on both sides of the sponge by electrospinning (Figure 15A). The multi-layer structural dressing was similar to the structure of natural skin but also had good physical properties, biocompatibility, permeability absorption and antibacterial ability, effectively promoting wound healing. Nonwoven fabrics have good flexibility and mechanical properties. By combining nonwoven fabrics with electrospun NFMs, the mechanical properties of dressings can be significantly improved. Qiu et al. [180] used zein and ethyl cellulose as basic materials to prepare the outer layer NFM containing an antibacterial agent and the inner layer NFM with a healing agent through electrospinning (Figure 15B). Then, nonwoven containing bacterial cellulose was used as the intermediate reinforcement layer and bonded under certain thermal pressure conditions. The existence of the intermediate layer compensated for the lack of mechanical properties of zein, while the addition of bacterial cellulose made it have good hygroscopicity and biocompatibility. In addition, the NFMs prepared from cellulose have a porous structure and good light transmittance, creating conditions for wound visualization to monitor wound changes better. Xia et al. [181] fabricated porous cellulose membranes with chitosan-coated nanofibers using a simple electrospinning technology (Figure 15C). The results showed that the composite membrane had high wettability, hydrophilicity, and gas permeability, in addition to excellent light transmittance and mechanical compliance. The influence of electrospun NFM structures on wound healing has been summarized in Table 4.

## 5. Conclusions

Electrospun nanofibers have a high specific surface area, high porosity, and a structure similar to the natural extracellular matrix of the skin, making them suitable and effective wound dressings. This review first provides an overview of the origin and development of electrospinning technology and analyzes its working principle and key process parameters. Afterwards, the influence of devices and materials for electrospinning on the morphology and properties of nanofibers is discussed. The advantages and limitations of electrospun matrix materials are described, the selection and combination of polymers are proposed, and the introduction of functional factors with hemostatic, antibacterial, cell proliferation and other therapeutic effects into electrospun matrix materials is illustrated. Then, the structural design of single nanofiber for wound dressing, including porous structure, bead structure, core-shell structure, and multicore-shell structure, is described in detail. Finally, a detailed introduction is given to the structural design of electrospun NFMs for wound dressings, containing ordered structure, double-layer biomimetic structure and multi-layer structure.

The structural design of single nanofiber and NFMs provides creativity for the production of more perfect wound dressings. However, the diversity of structures represents the complexity of the production process, such as the easy blockage of coaxial needles, fast volatilization of solvents in free surface electrospinning, and low mechanical properties of porous structured fibers, which increases the difficulty of selecting materials and limits the production efficiency of nanofibers to some extent. Moreover, the binding force between multi-layer NFMs or NFMs and other scaffolds also affects the overall stability of wound dressings. Nevertheless, as a simple and flexible method for preparing nanofibers, electrospinning technology still has great potential in the application of wound dressings. In recent years, electrospun nanofibers have been used to prepare functional yarns [182,183], which provides the possibility of using them as wound sutures, thus prompting electrospun nanofibers to be applied in more diverse forms in wound dressings. In the future, the preparation of electrospun nanofibers should avoid the use of toxic organic solvents and reasonably design electrospinning equipment to achieve its industrial development. The design of electrospun nanofibers will tend to meet more complex needs and adapt to wounds that are difficult to heal. For example, an NFM that can induce corresponding drug-release reactions according to the wound microenvironment or external stimulation will be developed to treat different types of wounds. Functional NFMs with diagnostic or therapeutic effects for wound treatment will be proposed. In general, wound dressings based on electrospun NFMs have a bright future due to their diversity in component selection and structural design.

## Figures and Tables

**Table 1:** Effects of parameters on electrospun nanofibers.

	Parameters	Effects
Process parameters	voltage	with increasing applied voltage, the fiber diameter first increases and then decreases [34]
	distance	too far, the electric field strength is significantly weakened, and the fiber diameter becomes larger
	flow rate	fast: the diameter of nanofibers gradually increases too fast: bead generation [35]
Solution parameters	polymer molecular weight	high: form uniform large-diameter fibers
	viscosity	too low: bead generation [36] high: the diameter of nanofibers gradually increases
	concentration	high: decrease in bead generation [37,38]
	conductivity	high: the nanofiber is stretched more fully, and its diameter is smaller [39]
	surface tension	too high: the fiber is prone to beaded structure or cannot be spun [40]
Environmental parameters	temperature	the fiber diameter will decrease with the increase in temperature [41]
	humidity	too high or too low humidity can increase the diameter [42,43]

**Table 2:** The influence of electrospun nanofiber composition design on wound healing.

Base Materials	Active Ingredients	Types	Functions	Ref.
polybutylene succinate/chitosan	/	blend	hemostatic	[101]
chitosan/PVA	tannic acid, zinc-based MOFs	blend	hemostatic	[102]
chitosan/PEO	kaolin	blend	hemostatic	[106]
PVA	AMX, MXene	blend	bacteriostatic	[111]
polyurethane	thymol	blend	antibacterial	[112]
chitosan/PEO	antibacterial peptides	blend	antibacterial	[114]
PCL/collagen	ZnO quantum dots	blend	antibacterial	[115]
PLGA/gelatin	rhEGF, gentamicin sulfate	blend	bacteriostatic, promoting the wound closure	[120]
collagen/GO	bFGF	blend	promote the wound closure	[121]
PVA	BMSCs	blend	promote the formation of granulation tissue and epithelialization	[125]
PLGA	LPS/IFN-gamma, BMSCs	blend	promote epithelialized regeneration, collagen remodeling and angiogenesis	[126]

**Table 3:** The influence of electrospun nanofiber structures on wound healing.

Base Materials	Active Ingredients	Structure	Functions	Ref.
cellulose acetate	thymol	porous	improve cell compatibility, promote cell proliferation	[131]
PCL	/	porous	promote the adsorption and growth of fibroblasts	[134]
PLA/CS	aloin	porous	good swelling property, excellent blood coagulability	[135]
PCL/PVA	curcumin	beaded	controlled release of drugs, excellent osmotic absorption ability	[144]
PVA, gelatin	advanced platelet-rich fibrin mixture	core-shell	high cell proliferation and adhesion rates	[148]
PCL, gelatin	ciprofloxacin, tetracycline hydrochloride	core-shell	antibacterial loading of the dual-drug system	[149]
PLA/polyurethane, PVP	/	hollow	high liquid absorption capacity	[150]
cellulose acetate, PCL	nisin	multicore-shell	bactericidal, high drug utilization rate	[152]
PCL, gelatin, PLGA	/	multicore-shell	excellent drug sustained release	[153]
PCL, chitin, silk	/	self-assembled multi-layer	antibacterial, angiogenesis and collagen deposition	[154]
silk/PCL, chitosan, collagen	/	self-assembled multi-layer	antibacterial, promote collagen deposition and reduce scar formation	[157]
PCL/collagen	transforming growth factor-β1	self-assembled multi-layer	promote angiogenesis, considerable fibroblast adhesion and diffusion ability	[160]

**Table 4:** The influence of electrospun NFM structures on wound healing.

Base Materials	Active Ingredients	Structure	Functions	Ref.
PCL	tazarantine	ordered	promote targeted cell growth and angiogenesis	[165]
PLLA	/	ordered	alleviate inflammation and promote angiogenesis	[166]
PLLA	dimethylglycan	porous and ordered	promote cell attachment, migration and revascularization	[167]
PCL, chitosan	copper-doped Laponite	double-layer	Antibacterial, promotes the migration of endothelial cells	[171]
PCL/PLA, PVA/chitosan/alginate	/	double-layer	promote cell proliferation, absorb excess tissue osmotic fluid	[172]
alginate, chitosan, PCL/collagen	doxycycline	multi-layer	shorten the inflammatory period, promote cell migration	[177]
chitosan/PVA, PCL	chamomile	multi-layer	good mechanical properties, bacteriostatic	[178]
zein/ethyl cellulose, bacterial cellulose	/	multi-layer	antibacterial, good hygroscopicity and biocompatibility	[180]

## Data Availability

All data generated or analyzed during this study are included in this published article.
